# Understanding quality of contraceptive counseling in the CHARM2 gender-equity focused family planning intervention: Findings from a cluster randomized controlled trial among couples in rural India^☆^

**DOI:** 10.1016/j.contraception.2022.10.009

**Published:** 2022-11-01

**Authors:** Sarah Averbach, Nicole E. Johns, Mohan Ghule, Anvita Dixit, Shahina Begum, Madhusudana Battala, Niranjan Saggurti, Jay Silverman, Anita Raj

**Affiliations:** a Center on Gender Equity and Health, University of California San Diego School of Medicine, La Jolla, CA, United States; b Department of Obstetrics, Gynecology, and Reproductive Sciences, University of California San Diego School of Medicine, La Jolla, CA, United States; c Department of Biostatistics, ICMR-National Institute for Research in Reproductive and Child Health, Mumbai, India; d Population Council, Zone 5A, India Habitat Center, New Delhi, India; e Department of Education Studies, Division of Social Sciences, University of California San Diego School of Medicine, La Jolla, CA, United States

**Keywords:** Contraceptive counseling, Family planning, Gender-transformative, Male engagement, Quality of care

## Abstract

**Objectives:**

The CHARM2 (Counseling Husbands and wives to Achieve Reproductive Health and Marital Equity) intervention engages health care providers to deliver gender-equity and family planning sessions to couples using a person-centered shared decision-making approach for contraception counseling. We previously showed that the intervention improved contraceptive use at 9-month follow-up. We sought to assess whether the intervention was further associated with the quality of care reported by participants and whether the quality of care reported mediated the effect of the intervention on contraceptive use.

**Study design:**

This is a planned secondary analysis of the effect of the CHARM2 intervention on 1201 married couples in rural Maharashtra, India in a cluster randomized controlled trial completed between 2018 and 2020. We assessed the effect of CHARM2 on perceived quality of care as measured by the Interpersonal Quality of Family Planning (IQFP) scale using a difference-in-differences linear regression approach including a mixed-effects model with nested random effects to account for clustering. We assessed whether the association between CHARM2 and modern contraceptive use was mediated by quality of family planning care.

**Results:**

Intervention participants had higher mean IQFP scores than control participants at 9-month follow-up (intervention 3.2, SD 0.6 vs. control 2.3 mean, SD 0.9, *p* < 0.001). The quality of care reported mediated the effect of the intervention on contraceptive use (indirect effect coefficient 0.29, 95% CI 0.07–0.50).

**Conclusion:**

Family planning interventions such as CHARM2, which utilize person-centered shared decision-making contraceptive counseling approaches improve women’s perceived quality of care. Effects on quality of care mediate observed effects of the intervention on contraceptive use.

**Implications:**

Contraceptive interventions should focus on improving person-centered outcomes, such as quality of care, rather than contraceptive use targets. By focusing on improving person-centered care, interventions will improve contraceptive use among those who desire a method while meeting the holistic reproductive health needs of clients and couples.

## 1. Introduction

Provision of person-centered, high-quality, contraceptive counseling has the potential to better meet the reproductive needs of women and couples and has been associated with increased contraceptive use [1–4] and greater satisfaction with counseling [5]. However, few intervention evaluations utilize person-centered outcome measures that assess the quality of contraceptive counseling and care provided [6]. Outcome measures in family planning that focus on contraceptive uptake may not adequately measure preferences for contraception use or nonuse [6–8]. Evaluation of family planning interventions in India have historically been reliant on contraceptive and fertility targets rather than outcome measures that assess whether clients’ reproductive preferences were met by the intervention [7].

Gender based power dynamics, including traditional gender norms, male control of and even violence against female partners, son preference, and gender-based inequality in decision-making in households, affect women’s reproductive agency (the capacity to enact choice) and contraceptive use in India [9–12]. Gender equity focused programs that engage men in contraceptive decision-making and address gender norms in contraceptive decision-making have the potential to improve women’s reproductive agency [13]. Gender equity focused family planning interventions have demonstrated effectiveness in improving contraceptive use in young married couples in India [14–16]. Only one of these interventions in India uses a person-centered shared decision-making model for contraception counseling, the Counseling Husbands to Achieve Reproductive Health and Marital Equity 2 (CHARM2) intervention. CHARM2 is a gender synchronized, gender-transformative family planning intervention for young married couples in rural India. CHARM2 was designed to engage men in, and to improve the quality of, family planning counseling using a shared decision-making model—where the clinician shares medical knowledge about contraception and the woman or couple shares their preferences, to arrive at a shared decision whether to use, or not use, contraception that is aligned with client preferences [17].

Recent evaluation of the CHARM2 intervention demonstrated significant improvements in couples’ contraceptive communication and women’s contraceptive agency or perceived ability to use a chosen method over 18-month follow-up, as well as a significant effect on contraceptive use at 9-month follow-up [16]. In this study, we expand the CHARM2 evaluation to determine its impact on women’s perceptions of quality of care, a preference-aligned outcome measure, and we explore whether quality of care mediates the previously observed intervention effects on other family planning outcomes. We hypothesized that the CHARM2 intervention would be associated with improved quality of counseling and that the impact of the intervention on contraceptive use would be mediated, at least in part, by the quality of contraceptive counseling provided.

## 2. Methods

This is an a priori planned secondary analysis of the CHARM2 study described elsewhere in detail [18]. The Institutional Review Boards of The University of California San Diego, the Indian Council of Medical Research - National Institute for Research in Reproductive and Child Health in India, and the Population Council approved the study. The study is registered at Clinical Trials Registry: number NCT03514914.

In this cluster randomized trial, 1201 young couples across 40 geographic clusters were randomized to the CHARM2 intervention or control condition (standard of care). Inclusion criteria for the CHARM2 study included married couples with women aged 18 to 29 years, both partners nonsterilized, living together for at least six months.

We recruited couples from the rural Pune district of Maharashtra, India from September 2018–June 2019 via random selection from household rosters and approached and interviewed them in their homes. We collected data at three time points: baseline, 9-month, and 18-month follow-up. At 9-month follow-up, 1089 women provided survey responses, full couple retention at nine months was 90.2% (Supplemental Fig. 1). At 18-month follow-up, 1088 women provided surveys; full-couple retention at 18 months was 90.5%. Data collection ended in December 2020.

Our independent variable was intervention condition. Couples in the intervention clusters received counseling sessions from CHARM2 providers between baseline and 9-month follow-up. Gender-matched health providers delivered two gender-equity and family planning counseling sessions with married husbands and wives separately in parallel, and a final session was delivered to the couple together, by either the male or female provider who delivered the individual sessions, whichever was available and/or preferred by the couple.

CHARM2 providers included public and private health providers within the couples’ geographic cluster area who were trained in the CHARM2 curriculum inclusive of gender equity and person-centered contraceptive counseling using a shared decision-making approach. Counseling sessions were supported by a desktop flip chart and contraceptive flash cards to facilitate patient-centered shared decision-making. Couples in the control condition were informed about local family planning services available at no cost from the public health sector. Control participants were not required to obtain contraceptive counseling or care.

**Fig. 1.**
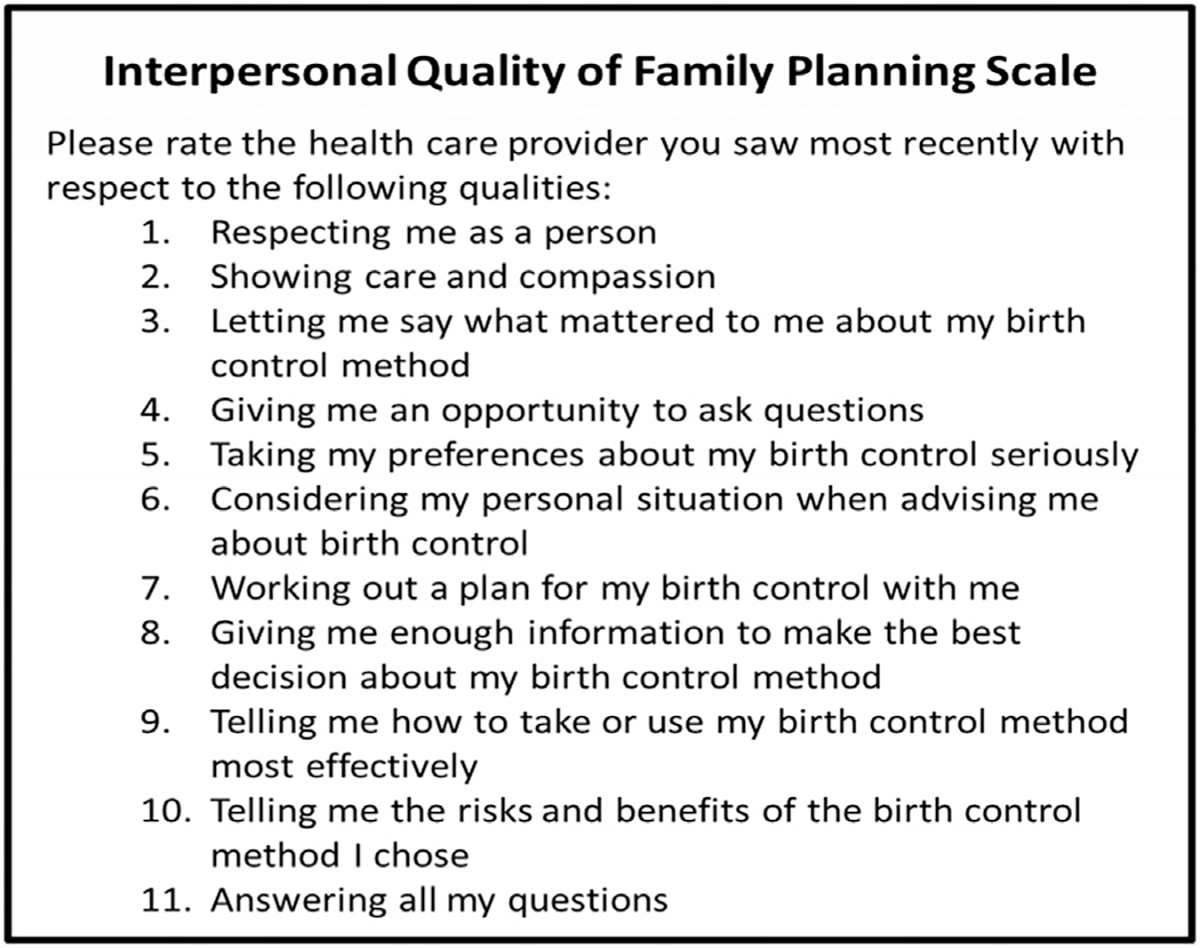
Interpersonal Quality of Family Planning (IQFP) items.

We utilized the Interpersonal Quality of Family Planning (IQFP) scale to measure our dependent variable, perceived quality of care. IQFP consists of 11 items assessing client perceptions of interpersonal connection, receiving adequate information, and decision support in their most recent family planning counseling (Fig. 1) [1]. Each item is a five-point Likert scale from poor (1) to excellent (5); the final score is an average of the 11 items with a range from 1 to 5, where higher score represents greater quality of care. Mean IQFP scores correlate to the following ratings: poor (1), fair (2), good (3), very good, (4) and excellent (5). We assessed the IQFP scale for all women who indicated that they had seen a family planning provider within the prior 9 months at each follow-up survey (at nine and 18 months).

### 2.1. Analysis

First, we assessed descriptive statistics including demographics and IQFP at each time point by intervention group. We utilized two-sided *t* tests for continuous variables, two-sided Fisher exact tests for binary variables and overall categorical variable distributions, and two-sided Wald tests for category-specific differences in categorical variables. Next, we assessed intervention effects on IQFP using a difference-in-differences linear regression approach including a mixed-effects model with nested random intercept specifications of individuals within subcenters (clusters) to account for cluster randomization. We constructed minimally adjusted models accounting only for time (baseline, 9-month follow-up, 18-month follow-up) and group and fully adjusted models accounting for baseline demographic characteristics associated with treatment group and/or study retention [16], including: wife age (continuous), husband age (continuous), wife age at marriage (continuous), parity (0, 1, 2, or more), religion (Hindu or non-Hindu), caste (Scheduled Caste/Scheduled Tribe/Other Backwards Class [official designation in India, reflects socioeconomic disadvantage] and general [official designation for caste which is not of the prior three types]), below poverty line (BPL) card ownership [a proxy for low income] (yes/no), living son (yes/no), and coresidence with mother-in law (yes/no).

We evaluated the IQFP scale as a continuous mean score and as a categorical average response in descriptive analyses, and as a continuous mean score only in unadjusted and adjusted regression analyses. We recorded the mean score as “Poor/Fair” (average score 1–2.5), “Good” (average score 2.51–3.5), and “Very good/Excellent” (average score 4.51–5) for the categorical response.

To assess potential mediation of improved quality of family planning care on other observed CHARM2 treatment effects [16], we conducted a series of mediation analyses for wife’s reports of: modern contraceptive use, contraceptive communication, self-efficacy, and equal right to decide to use contraception as husband. We used 9-month data for mediation analyses, to most directly capture family planning counseling delivered by CHARM2 providers (at 18 months, CHARM2 participants may have sought family planning care from additional providers who did not participate in the CHARM2 intervention). All analyses were conducted using STATA 15.1.

Assuming a baseline average IQFP score (2.62) [19] we estimated that a 20% relative increase from baseline, or an increase of 0.52 points would be clinically meaningful. Using baseline data, we assumed 40% of women would report seeing a provider and answer all IQFP items, a standard deviation in IQFP score of 0.94, and an intra-cluster correlation coefficient of 0.15. Using a two-sided test accounting for clustering, we estimated that our expected sample size would provide 81% power to detect that difference with an alpha of 0.05.

## 3. Results

At baseline, 504 of 1201 women (42%) reported seeing a family planning provider in the previous 9 months; 38% of intervention and 46% of control participants (*p* = 0.002). At 9-month follow-up, 723 of 1089 women (66%) had seen a family planning provider in the prior nine months; 92% of intervention and 42% of control participants (*p* < 0.001). At 18-month follow-up, which was conducted following the first nationwide shutdown for COVID-19 in 2020, only 213 of 1088 women (20%) had seen a family planning provider in the prior nine months, 19% of intervention and 20% of control participants (*p* = 0.77) (Supplemental Fig. 1).

All five counseling sessions were received by 87.5% of couples in the intervention arm; an additional 7.3% of women and 5.3% of men received at least one session. Only 3.0% of couples in the intervention arm received no sessions.

**Fig. 2.**
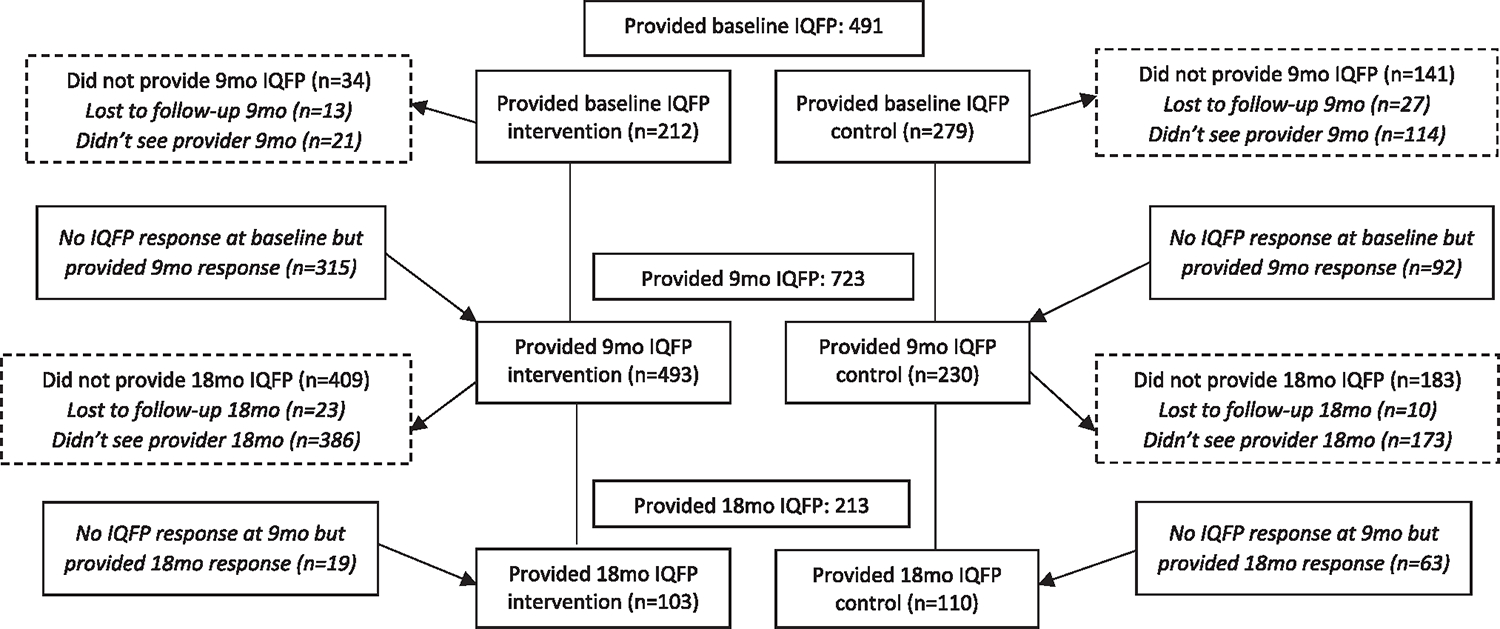
Female participant IQFP item response by CHARM2 intervention group (*n* = 948) Maharashtra, India 2018–2020.

Among women who reported seeing a family planning provider in the prior 9 months, most provided responses to all IQFP items; 491 of 504 at baseline (97%), 723 of 724 at 9-month (99%), and 213 of 213 at 18-month follow-up (100%) (Fig. 2). Women who provided IQPF scores at any point had had husbands who were one year younger on average (29 vs. 30 at baseline, *p* = 0.03). There were no other demographic differences between those who provided IQFP and those who did not.

Thus, the final analytic sample for this study includes 491 women at baseline, 723 women at 9-month follow-up, and 213 women at 18-month follow-up; 948 unique women providing data at one time point at least, with 55 women providing data at all three time points. Women in the intervention group were more likely to be Hindu than women in the control group (96.9 vs. 85.2%, *p* < 0.001). There were no other statistically significant differences between groups (Table 1).

At baseline, the average IQFP score was 2.6 out of 5 (SD 0.9), equivalent to a rating between “fair” and “good” (Table 2). Average IQFP scores did not differ significantly between intervention (mean 2.5, SD 1.1) and control (mean 2.7, SD 0.8) participants (*p* = 0.09) at baseline. However, intervention participants were more likely to rate their last provider before the intervention negatively (poor/fair) compared to control participants (55% vs. 42%, *p* = 0.004) and less likely to rate their provider as “good” than control participants (26% vs. 43%, *p* < 0.001).

**Fig. 3.**
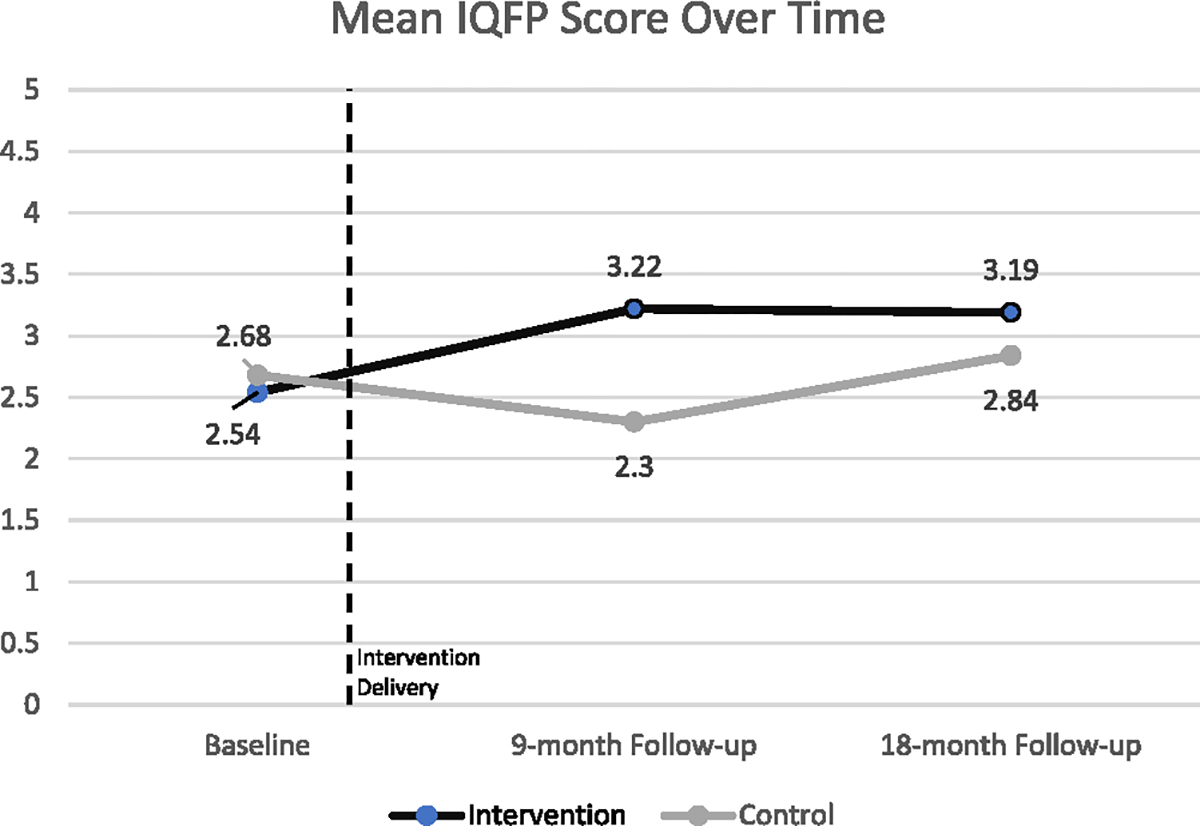
Mean Interpersonal Quality of Family Planning (IQFP) score over time, by treatment group (*n* = 948) Maharashtra, India 2018–2020.

At 9-month follow-up, the average IQFP score was 2.9 out of 5 (SD 0.8), equivalent to a rating of approximately “good.” Intervention participants had significantly higher mean IQFP scores than control participants at 9-month follow-up (intervention mean 3.2, SD 0.6 vs. control mean 2.3, SD 0.9, *p* < 0.001) (Fig. 3). Intervention participants were nearly three times *more* likely to rate their provider highly (very good/excellent; 26% vs. 9%, *p* < 0.001) and were nearly 10 times *less* likely to rate their provider negatively (poor/fair; 6% vs. 58%, *p* < 0.001).

At 18-month follow-up, the average IQFP score was 3.0 out of 5 (SD 0.7), equivalent to a rating of “good.” Intervention participants had significantly higher mean IQFP scores than control participants at 18-month follow-up (intervention mean 3.2, SD 0.6 vs. control mean 2.8, SD 0.7, *p* < 0.001). Intervention participants were *half* as likely to rate their provider negatively (poor/fair; 15% vs. 29%, *p* = 0.01).

In minimally adjusted models (accounting for time, treatment status, and geographic cluster), intervention relative to control condition was associated with a one-point increase in IQFP score from baseline at 9-month follow-up (1.05, 95% CI 0.61–1.48, *p* < 0.001) and a half-point increase from baseline at 18-month follow-up (0.49, 95% CI 0.07–0.92, *p* = 0.02) (Table 3). These associations were maintained when demographic characteristics were included (9-month: 1.04, 95% CI 0.62–1.47, *p* < 0.001; 18-month: 0.52, 95% CI 0.08–0.95, *p* = 0.02). In this adjusted model, only having one child at baseline, relative to no children at baseline, was significantly associated with IQFP score (0.14, 95% CI 0.004–0.27, *p* = 0.04); wife age, husband age, wife age at marriage, having a living son, religion, caste, poverty, and coresidence with mother-in law were not associated with IQFP score at *p* < 0.05.

In analyses assessing mediation of CHARM2 treatment effects by improved QOC at 9-month follow-up, we found evidence of significant mediation of the treatment effect on current modern contraceptive use (indirect effect coefficient 0.29, 95% CI 0.07–0.50). We did not observe statistically significant mediation of any other assessed outcome including wife’s reports of contraceptive communication, self-efficacy, and equal right to decide to use contraception as her husband (data not shown).

## 4. Discussion

We found that the CHARM2 intervention had a significant effect on women’s perceptions of interpersonal quality of care received from their family planning providers and that this effect on quality of care mediated observed effects of the CHARM2 intervention on contraceptive use. Interestingly, quality of care did not demonstrate mediation effects on contraceptive communication and contraceptive self-efficacy outcomes; these outcomes may be more attributable to engagement of men in the CHARM2 intervention rather than the quality of care women report. However, our study design does not allow us to compare male engagement components to women’s person-centered counseling components. These findings indicate the value of the person-centered shared decision-making approach utilized in CHARM2 to support quality of care in family planning counseling for women and that this care can increase women’s uptake of contraceptive use, corresponding to prior evidence showing an association with person-centered care and contraceptive use [1–4].

We found low mean IQFP scores for both groups at baseline, and for the control group throughout the study, highlighting the need for more person-centered counseling methods, like CHARM2, in this population. Our findings support the capacity for health systems to support person-centered care for women while engaging men in family planning counseling simultaneously, with gender synchronized sessions. Concerns regarding male partner engagement as compromising female reproductive agency [20] can be addressed via person-centered care for women and inclusion of gender equity focus. High quality interpersonal communication could be a mechanism that allows for identification of how and when men should be engaged in contraceptive decision-making for each client. More research is needed to understand which components of the intervention resulted in the observed impacts [21]. Nonetheless, our study highlights that interventions should be designed to improve quality of care, rather than specific contraceptive use targets, and that quality of care should be a standard evaluation outcome in family planning related intervention research.

Our study has several limitations. Outcomes were reliant on self-report and collected by interview, and are, therefore, vulnerable to recall and social desirability biases. High follow-up rates (> 80%) reduce follow-up biases, though the sample is reduced in analyses restricted to women who reported receiving family planning care in the prior 9 months. At 9-month follow-up, all women in the intervention arm who received at least one intervention session (95% of intervention participants) had in fact seen a family planning provider in the prior 9 months; however, this item was asked directly of women and 33 women who received sessions reported they had not seen a provider and did not provider IQFP scale responses. This may be due to issues of recall or misunderstanding the question.

Another limitation is that we cannot ascribe the quality of care outcomes to CHARM2 intervention participation with certainty (respondents were not asked to name the provider for whom they were rating quality of care). Treatment condition respondents would most likely have seen a CHARM2 provider in the prior 9 months at 9-month follow-up due to the nature and timing of the intervention, and would likely revisit that provider given an established relationship, physical proximity, free services, and continuity of care, particularly if perceived quality of care was high. Additionally, observed intervention effects, particularly at 18-month follow-up, may be attributable to women more effectively engaging with providers subsequent to CHARM2 receipt, whether or not the provider was trained in the CHARM2 intervention. Findings from this study have limited generalizability, to the region of India in which we worked and to rural young married couples. Nonetheless, findings may inform efforts to increase person-centered contraceptive care and use of preference-aligned outcome indicators to assess intervention effects in other contexts and populations, particularly within India. While our rigorous cluster randomized controlled trial design is a strength of the study, multiple arms to compare intervention components would offer greater insight into the mechanisms by which the intervention led to significant improvements in outcomes. Data collection at follow-up encompassed the period of the COVID-19 pandemic and subsequent lockdown periods in India, and the pandemic may have affected access to contraceptives as well as women’s agency [22, 23], potentially affecting our study outcomes at 18-month follow-up. Longer-term follow-up after the pandemic will offer greater insight into potential sustained intervention effects. Finally, we did not utilize the 4-item person centered contraceptive counseling (PCCC) scale since the parsimonious version of the scale had not been validated when we designed this study. However, the shorter form PCCC is a reliable outcome measure to consider for future validation and use in global settings [24].

In summary, gender equity focused family planning interventions such as CHARM2, which utilize person-centered care models improve women’s perceived quality of care. Effects on quality of care mediate the observed effects of the intervention on contraceptive use. These findings highlight the value of shared decision-making counseling strategies to support male engagement interventions while still prioritizing women’s reproductive agency and the importance of utilizing preference-aligned outcome measures to assess the effect of family planning interventions.

## Supplementary Material

Supplement CHARM2 Averbach Contraception

## Figures and Tables

**Table 1:** Baseline characteristics of CHARM2 intervention participants who provided IQFP scale response for at least one time point (*n* = 948) Maharashtra, India 2018–2020

	Overall	Intervention	Control

*N*	*948*	*543*	*405*
Wife age (years), mean (SD)	24 (2.9)	24 (2.9)	24 (2.9)
Husband age (years), mean (SD)	29 (3.7)	29 (3.8)	29 (3.7)
Wife age at marriage (years), mean (SD)	19 (2.3)	19 (2.3)	19 (2.4)
Wife parity			
0	162 (17.1%)	94 (17.3%)	68 (16.8%)
1	496 (52.3%)	286 (52.7%)	210 (51.9%)
2 +	290 (30.6%)	163 (30.0%)	127 (31.4%)
Religion			
Hindu	871 (91.9%)	526 (96.9%)	345 (85.2%)
Non-Hindu	77 (8.1%)	17 (3.1%)	60 (14.8%)
Caste			
General	636 (67.1%)	364 (67.0%)	272 (67.2%)
Scheduled Caste/Scheduled	312 (32.9%)	179 (33.0%)	133 (32.8%)
Tribe/Other Backwards Class			
Household has below poverty			
line card			
No	717 (75.7%)	413 (76.2%)	304 (75.1%)
Yes	230 (24.3%)	129 (23.8%)	101 (24.9%)
Has living son			
No	502 (53.0%)	290 ( 53.4%)	212 (52.3%)
Yes	446 (47.0%)	253 (46.6%)	193 (47.7%)
Mother-in-law lives in same household			
No	190 (20.0%)	99 (18.2%)	91 (22.5%)
Yes	758 (80.0%)	444 (81.8%)	314 (77.5%)

**Table 2:** Interpersonal Quality of Family Planning score by treatment group and time among women participating in the CHARM2 intervention in rural India (n = 948) Maharashtra, India 2018–2020

	Baseline				9-month follow-up				18-month follow-up			
	Overall	Intervention	Control	*p* *	Overall	Intervention	Control	*p* *	Overall	Intervention	Control	*p* *

*N*	491	212	279		723	493	230		213	103	110	
Average score [mean (SD)]				0.09				<0.001				<0.001
Score, range 1–5	2.62 (0.94)	2.54 (1.06)	2.68 (0.83)		2.92 (0.79)	3.22 (0.55)	2.30 (0.87)		3.01 (0.68)	3.19 (0.63)	2.84 (0.67)	
Categorical average score [*n* (%)]				<0.001				<0.001				0.02
Poor/fair	232 (47.3%)	116 (54.7%)	116 (41.6%)	0.004	165 (22.8%)	31 (6.3%)	134 (58.3%)	<0.001	47 (22.1%)	15 (14.6%)	32 (29.1%)	0.01
Good	175 (35.6%)	54 (25.5%)	121 (43.4%)	<0.001	410 (56.7%)	335 (67.9%)	75 (32.6%)	<0.001	117 (54.9%)	59 (57.3%)	58 (52.7%)	0.50
Very good/excellent	84 (17.1%)	42 (19.8%)	42 (15.0%)	0.17	148 (20.5%)	127 (25.8%)	21 (9.1%)	<0.001	49 (23.0%)	29 (28.2%)	20 (18.2%)	0.08

* Intervention vs. control; *t* test for average score, Fisher exact test for overall categorical score, Wald test for specific categories of categorical score.

**Table 3:** Mixed effects linear regression difference-in-differences models of average IQFP score (*n* = 948) Maharashtra, India 2018–2020

	Simple		Adjusted	
	*B*	95% CI	*B*	95% CI

Treatment				
Control cluster	0	[0.00, 0.00]	0	[0.00, 0.00]
Intervention cluster	−0.12	[−0.48, 0.23]	−0.13	[−0.48, 0.22]
Time				
Baseline	0	[0.00, 0.00]	0	[0.00, 0.00]
9-month follow-up	−0.38**	[−0.62, −0.13]	−0.36**	[−0.61, −0.11]
18-month follow-up	0.15	[−0.14, 0.43]	0.15	[−0.15, 0.45]
Time-treatment interaction				
Intervention # 9-month follow-up	1.05***	[0.61, 1.48]	1.04***	[0.62, 1.47]
Intervention # 18-month follow-up	0.49*	[0.07, 0.92]	0.52*	[0.08, 0.95]
Wife age (years)	-	-	0.01	[−0.01,0.04]
Husband age (years)	-	-	−0.01	[−0.02, 0.01]
Wife age at marriage (years)	-	-	0.01	[−0.01, 0.03]
Wife parity				
0	-	-	0	[0.00, 0.00]
1	-	-	0.14*	[0.00, 0.27]
2 +	-	-	0.13	[−0.06, 0.31]
Religion				
Hindu	-	-	0	[0.00, 0.00]
Non-Hindu	-	-	−0.02	[−0.14, 0.10]
Caste				
General	-	-	0	[0.00, 0.00]
Scheduled Caste/Scheduled Tribe/Other	-	-	−0.05	[−0.14, 0.05]
Backwards Caste				
Household has below poverty line card	-	-	−0.01	[−0.13, 0.11]
Has living son	-	-	0.07	[−0.01, 0.16]
Mother-in-law lives in same household	-	-	0.07	[−0.01, 0.16]

* p < 0.05.

** p < 0.01.

*** p < 0.001.
