# Supplementary material for: Understanding quality of contraceptive counseling in the CHARM2 gender-equity focused family planning intervention: Findings from a cluster randomized controlled trial among couples in rural India
Source: Contraception. Author manuscript; Available in PMC 2023 Dec 4. (PMC10695301; doi:10.1016/j.contraception.2022.10.009)
Supplement: Supplement CHARM2 Averbach Contraception [file NIHMS1943705-supplement-Supplement_CHARM2_Averbach_Contraception.docx]

**Supplemental Figure 1.** Female participant follow up and Interpersonal Quality of Family Planning (IQFP) item response by CHARM2 intervention group (N=1201), Maharashtra India, 2018-2020

*Additional Lost to follow-up 18mo (n=27)*

*Additional Lost to follow-up 18mo (n=18)*

Provided 18 month IQFP (n=110)

Provided 9 month IQFP (n=230)

Provided 18 month IQFP (n=103)

Provided 9 month IQFP (n=493)

*Regained at 18mo (n=18)*

*Regained at 18mo (n=26)*

Provided baseline IQFP (n=212)

Enrolled: 1201

Surveyed baseline: 1201

Surveyed 9 months: 1089

Surveyed 18 months: 1088

Enrolled intervention (n=600)

Received full intervention (n=525)

Received partial intervention (n=57)

Did not receive intervention (n=18)

Enrolled control (n=601)

Surveyed baseline intervention (n=600)

Surveyed baseline

control (n=601)

*Lost to follow-up 9mo (n=63)*

*Lost to follow-up 9mo (n=49)*

Followed up 9 months (n=537)

Complete couples: 532

Female survey only: 5

Followed up 9 months (n=552)

Complete couples: 551

Female survey only: 1

Followed up 18 months (n=536)

Complete couples: 535

Female survey only: 1

Followed up 18 months (n=552)

Complete couples: 552

Female survey only: 0

Provided baseline IQFP (n=279)
